# Fecal Microbiota Transplantation (FMT) as an Adjunctive Therapy for Depression—Case Report

**DOI:** 10.3389/fpsyt.2022.815422

**Published:** 2022-02-17

**Authors:** Jessica P. K. Doll, Jorge F. Vázquez-Castellanos, Anna-Chiara Schaub, Nina Schweinfurth, Cedric Kettelhack, Else Schneider, Gulnara Yamanbaeva, Laura Mählmann, Serge Brand, Christoph Beglinger, Stefan Borgwardt, Jeroen Raes, André Schmidt, Undine E. Lang

**Affiliations:** ^1^Department of Psychiatry (UPK), University of Basel, Basel, Switzerland; ^2^Department of Microbiology and Immunology, Rega Institute for Medical Research, KU Leuven-University of Leuven, Leuven, Belgium; ^3^Center for Affective, Stress- and Sleep Disorders (ZASS), Psychiatric Clinics (UPK), University of Basel, Basel, Switzerland; ^4^Sleep Disorders Research Center, Kermanshah University of Medical Sciences, Kermanshah, Iran; ^5^Substance Abuse Prevention Research Center, Kermanshah University of Medical Sciences, Kermanshah, Iran; ^6^Division of Sport Science and Psychosocial Health, Department of Sport, Exercise, and Health, University of Basel, Basel, Switzerland; ^7^Department of Psychiatry, School of Medicine, Tehran University of Medical Sciences, Tehran, Iran; ^8^Department of Research, St. Clara Hospital, Basel, Switzerland; ^9^Department of Psychiatry and Psychotherapy, University of Lübeck, Lübeck, Germany

**Keywords:** FMT, depression, gastrointestinal, microbiome-gut-brain axis (MGBA), case report

## Abstract

Depression is a debilitating disorder, and at least one third of patients do not respond to therapy. Associations between gut microbiota and depression have been observed in recent years, opening novel treatment avenues. Here, we present the first two patients with major depressive disorder ever treated with fecal microbiota transplantation as add-on therapy. Both improved their depressive symptoms 4 weeks after the transplantation. Effects lasted up to 8 weeks in one patient. Gastrointestinal symptoms, constipation in particular, were reflected in microbiome changes and improved in one patient. This report suggests further FMT studies in depression could be worth pursuing and adds to awareness as well as safety assurance, both crucial in determining the potential of FMT in depression treatment.

## Introduction

Major depressive disorder (MDD) is an illness affecting more than 264 million people worldwide (1) and influencing functioning and quality of life (QoL) (2). Despite advancements in the development of therapeutics, current treatments have not reached optimal efficacy and approximately one third of patients do not respond to treatment after two or more trials of antidepressant medication (3, 4). Therefore, the identification of new treatment options is crucial.

Recently, interest has been drawn toward the importance of the biochemical signaling between the gastrointestinal (GI) and the central nervous system, also known as the microbiome-gut-brain axis (MGBA) (5–9). Several studies have linked the gut microbiome to depression (6, 8–11). The gut microbiota composition appears to be altered in depressed people (12–15), presenting predominance of potentially harmful bacterial groups and/or reduction in beneficial bacterial groups (12). Such dysbiosis could be related to depressive symptoms (16, 17), as the MGBA is a bi-directional pathway, which involves multiple communication modalities, including metabolites, the immune system or the vagus nerve (8, 10, 18, 19). Together, these studies feed the hypothesis that modification of the gut microbiome could decrease MDD symptoms. There are various ways to manipulate the gut microbiome, such as administration of prebiotics (20–22), probiotics (21, 22), postbiotics (22), or fecal microbiome transplantation (FMT) (23). Preclinical evidence showed that adult germ-free rodents receiving fecal samples from MDD-patients showed increased depressive-like behavior compared to controls (17, 23). Therefore, transplanting healthy fecal microbiome to MDD-patients could potentially ameliorate depressive symptoms. The intention of FMT is to introduce a beneficial microbial gut community by transferring intestinal microbiota from a healthy donor to a patient.

FMT has proven to be an effective treatment for recurrent *Clostridium difficile infection* (*rCDI*) (24, 25). To our best knowledge, in MDD only one case treated with FMT has been reported so far (26).

Therefore, we initiated a randomized controlled trial (RCT), testing the efficacy of oral frozen FMT-capsules as adjuvant therapy in patients with moderate/severe MDD at the University Psychiatric Clinics Basel (UPK). While our RCT was running, the Food and Drug Administration released a safety alert regarding FMT (see Supplementary Methods for details) and we decided to cease the study for safety reasons after including a total of four patients. In the present article, we report clinical and safety results from two cases that had already received the active product.

## Case Descriptions

**Figure 1:**
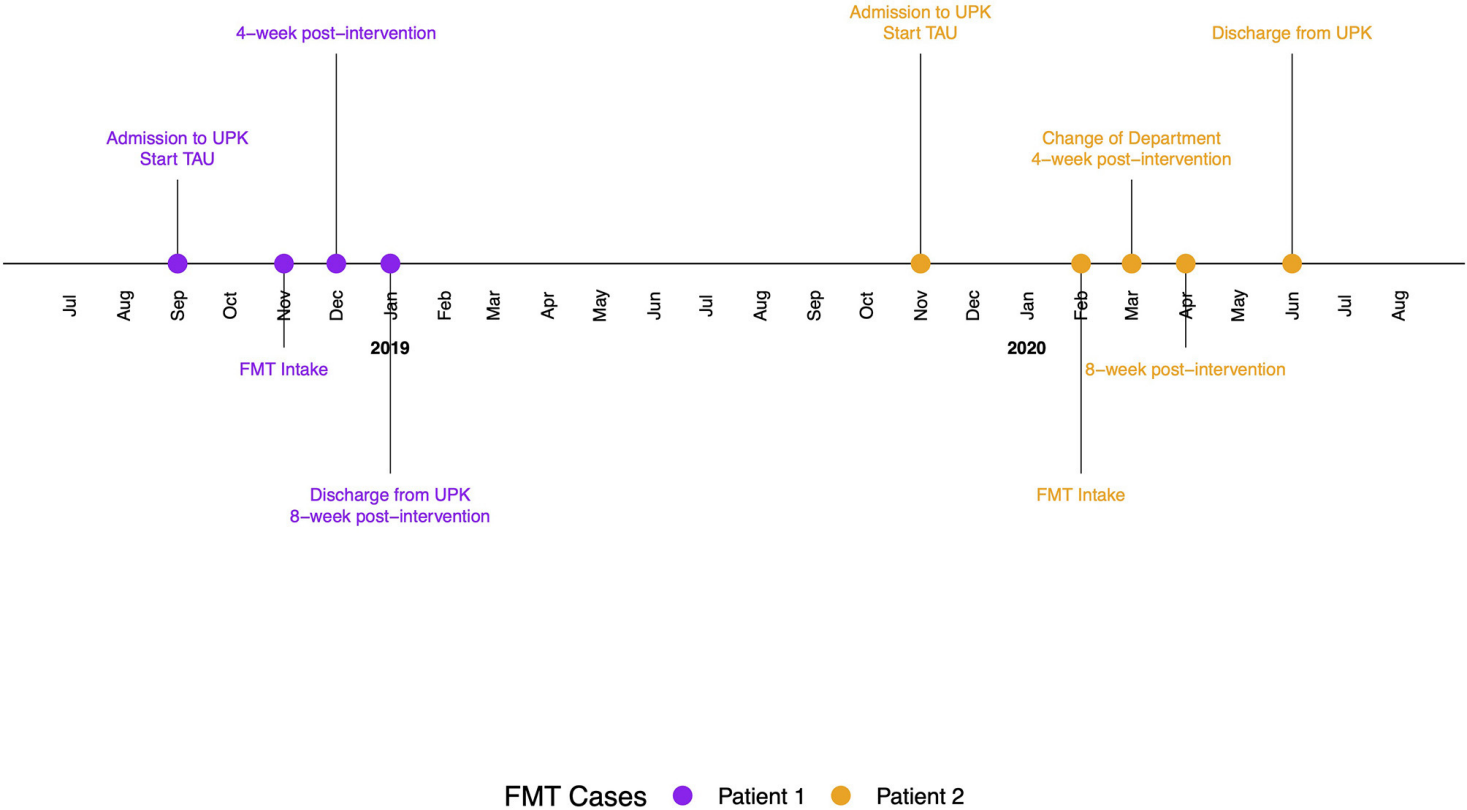
Timeline with relevant timepoints from the episode of study and care.

Both patients were women and between 50 and 60 years old (Table 1). Before starting the intervention, both received treatment as usual (TAU), which included pharmacological treatment, psychotherapy and additional medical-therapeutical treatments (Figure 1 and Supplementary Tables 1, 2. Changes in their treatment took place based on medical indication and patients' preferences.

**Table 1:** Sociodemographic information.

	**Patient 1**	**Patient 2**
Age (years)	53	58
Body-mass index (kg/m^2^)	29.3	37.1
Sex	Female	Female
Ethnicity	Caucasian	Caucasian
Years with diagnosed depression	12	39
Number of hospitalizations	2	2
Marital status	Unmarried	Married
Gastrointestinal problems	Constipation, stomach pain, bloating, burping, sickness	Constipation, flatulence
Hamilton depression scale (HAMD), sum score	21	31
Gastrointestinal symptom-rating scale (GSRS), sum score	79	30

Patient 1 had a diagnosis of MDD and chronic constipation. According to the patient, her first depressive episode started in adolescence, with a suicide attempt later in life. She was diagnosed with MDD in 2006 and had been hospitalized twice in her life. Depression is common in her family, with two male second-degree relatives having suffered from it, one deceased by suicide. At the time of baseline assessment, the patient had been in therapy as an inpatient for almost 3 months and treated for depression and constipation with persevering symptoms (see Supplementary Table 1).

Patient 2 had a diagnosis of MDD. She was diagnosed with depression in 1980 and has been hospitalized twice since then. A family history of depression is unknown. After more than 2 months of inpatient treatment, the patient's symptoms persevered. She was medicated with antidepressants and benzodiazepines (Supplementary Table 2). At time of FMT intervention, the patient was suffering from negative emotions and GI symptoms, such as flatulence and constipation. For a more detailed description of the patients (see Supplementary Case Description).

## Methods

### Diagnostic Assessment and Study Design

The RCT was approved by the local ethics committee (Ethikkommission Nordwest- und Zentralschweiz) and was conducted in accordance with the principles of the Declaration of Helsinki and the International Conference on Harmonization Tripartite Guideline on Good Clinical Practice. Eligible patients were informed about the study and provided written informed consent. The study was registered at ClinicalTrials.gov prior to study start (NCT03281004). MDD-patients were recruited from the UPK (Switzerland). At baseline, we assessed depressive symptoms [Hamilton Depression Rating Scale; HAMD (27) and Beck Depression Inventory-II; BDI II (28)], GI-symptoms [Gastrointestinal Rating Scale; GSRS (29)] and collected anthropometric and demographic data. Then the intake of oral frozen FMT-capsules followed. After the treatment, participants were observed and assessed on a weekly basis over a period of 4 weeks. After 4 weeks, post-intervention measurements were conducted. Additionally, an 8-week follow-up was performed. Stool samples were collected at baseline and 4 weeks after the intervention. For one of the patients, stool samples were available 8 weeks after the intervention (Supplementary Material).

### Intervention

Patients were administered 30 oral frozen FMT-capsules within 90 min under the observation of a physician. Each active 30-capsule-dose consisted of 8.25 g donor stool, originating from a single donor, which was a different donor for each patient. For a detailed description of the methods (see Supplementary Methods).

## Outcomes

Both patients adhered to the intervention. They tolerated the oral frozen FMT-capsules well and did not report any serious adverse events (SAEs).

### Depressive Symptoms

**Figure 2:**
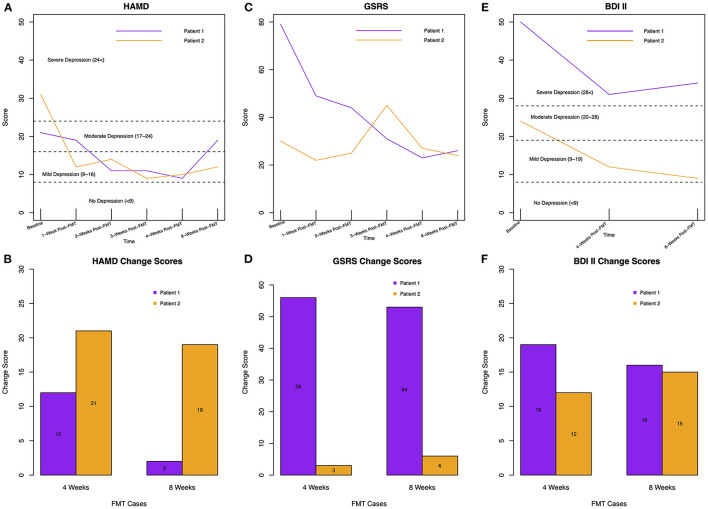
**(A)** HAMD-scores for patient 1 and 2 over time, including cut-offs for depressive symptom severity. **(B)** Change scores of HAMD rating for both patients at 4-weeks compared to baseline and 8-weeks compared to baseline. A higher (and positive) change score indicates improvement of depressive symptoms as the change score was calculated by subtracting the score at post-intervention from the score at baseline (e.g., baseline score: 21, post-intervention score: 9, equals 21–9 = 12). **(C)** GSRS-scores for both patients over time; without cut-off for GI-symptom severity as different clusters of symptoms are defined by the GSRS (e.g., constipation) and classification of severity would be only possible for each symptom, not for the overall score. **(D)** GSRS change scores for both patients at 4-weeks compared to baseline and 8-weeks compared to baseline. **(E)** BDI-II-scores for both patients over time. **(F)** BDI-II change scores for both patients at 4-weeks compared to baseline and 8-weeks compared to baseline.

For patient 1, symptoms of depression improved, indicated by a decreased HAMD-score from 21 points at baseline to nine points 4 weeks post-intervention. At 8-week follow-up, the HAMD-score increased to 19 points (Figure 2A). For patient 2, the HAMD-score decreased from 31 to 10 points after 4 weeks and increased by two points after 8 weeks (Figure 2A). Thus, the FMT intervention resulted in a 4-week change score of 12 and 21 HAMD-points for patient 1 and 2, respectively, and an 8-week change score of 2 and 19 points, respectively (Figure 2B). As the present results come from two cases and do not allow for statistical comparison, we mapped our HAMD change scores together with meta-analysis results from Kirsch et al. (30) (Supplementary Figures 1, 2); methods and results can be found in the Supplementary Material. Additionally, we explored the depressive symptoms from two patients, who had received placebo. Both placebo-patients tolerated the placebo-capsules well. HAMD-scores from both placebo-patients only improved within the first 2 weeks after placebo-intake and increased back to baseline scores, which is visible in Supplementary Figures 3A,B (placebo 1: baseline 16, post-intervention 16, follow-up 9; placebo 2: baseline 22, post-intervention 22, follow-up not available).

The BDI-II-scores dropped for both FMT-patients 4 weeks after FMT; from 50 to 31 points for patient 1 and from 24 to 12 points for patient 2, which results in change scores of 19 and 12, respectively. At 8-week follow-up, patient 1 reported a BDI-II-score of 34 and patient 2 a score of 9, resulting in change scores from baseline to follow-up of 16 and 15, respectively (Figures 2E,F).

### Gastrointestinal Symptoms

Gastrointestinal symptoms improved in both patients (Figure 2C). Patient 1 benefitted by 56 points after 4 weeks, which worsened by only three points after 8 weeks. At baseline, she suffered from stomach pains, sickness, bloating, burping, and constipation. All symptoms improved over time. Patient 2 reported only a slight improvement of three points 4-week post-FMT and continued reporting constipation and bloating. The patient slightly improved by three more points at 8-week follow-up (Figures 2C,D).

### Microbiome Composition and Diversity

**Figure 3:**
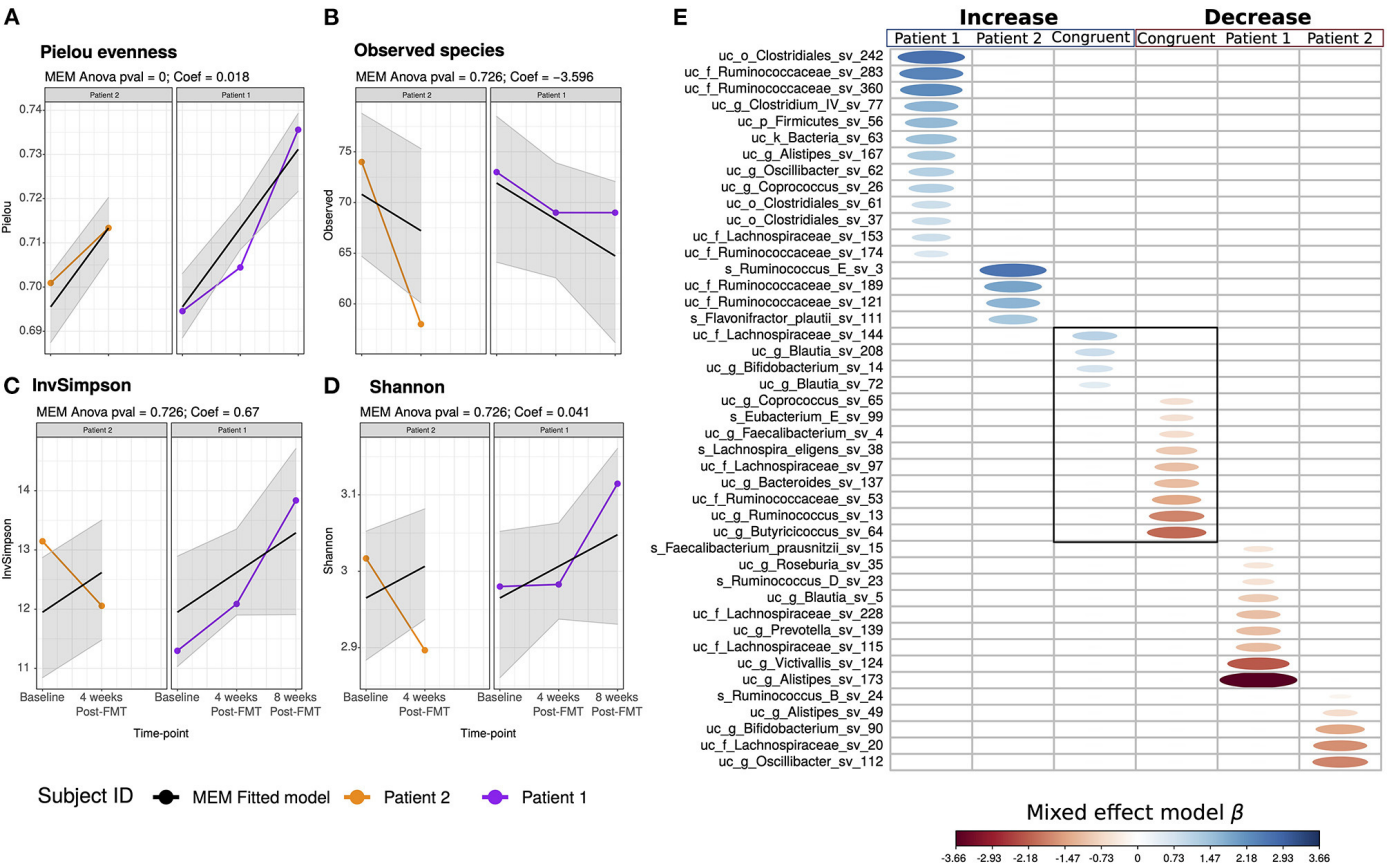
Patients' microbiome diversity. Mixed-effects models (MEM) of the genus level **(A)** Pielou evenness, **(B)** observed species, **(C)** inverse simpson, and **(D)** Shannon index, the alpha diversity estimators were modeled into a discrete manner and represent its results as boxplot and into a continuous way, representing the MEM slope into line-plots. The gray area into the continuous MEM represents the 95% confidence level. Patient 1 is displayed in purple, patient 2 in orange. **(E)** Mixed-effects models (MEM) of the ASV after the FMT intervention. The columns remarked in blue indicates the increase in the abundance of the ASV of patient 1, patient 2, and when the increase is congruent between both. Similarly, the columns remarked in blue indicates the decrease of the ASV in time. The blue scale represents the MEM positive coefficient; in red, the negative. The ASV was set as a putative biomarker if both the continuous and discrete mixed-effect-model time coefficients were significant (ANOVA < 0.05).

In both patients, moisture decreased over time [Supplementary Figure 6A; Mixed-effects model (MEM) ANOVA *p*_*adjusted*_ < 0.05]. Our patients showed lower moisture levels than the ones reported in a healthy population (31) (Wilcoxon test: W = 50, *p* = 0.025, Supplementary Figure 7). The bacterial load reflected in the cell counts was constant among the three time points for both patients (MEM ANOVA *p*_*adjuste*_*d* = 1). Diversity analysis was done to measure the taxonomic evenness (Pielou index), the richness (the number of observed species) and the diversity (inversed Simpson and Shannon indices) after FMT. Both patients showed an increase in the Pielou index after FMT, suggesting an evolution toward a more even distribution of species (Figure 3A). The species richness was generally reduced upon intervention (Figure 3B) and sustained until week eight in patient 1 (Figure 3B). We found that patient 1 showed increased inversed Simpson and Shannon diversity (Figures 3C,D), whereas patient 2 showed decreased diversity (Figures 3C,D).

The FMT intervention revealed a different effect on the bacterial composition of the two patients. Patient 1 maintained the Ruminococcus enterotype over all time points; while patient 2 switched from the Ruminococcus to the Bacteroides-2-enterotype 4 weeks post-intervention (Supplementary Figure 8), indicating a dysbiotic microbial composition previously linked to fast transit and inflammation (31, 32). The switch in the bacterial composition was also associated with an increase of the fecal calprotectin levels, which was higher than the ones reported in healthy subjects (Supplementary Figure 9A) (31). However, the bacterial load increased in patient 2 4 weeks post-intervention; the cell counts increased to a level between the 75 and 95 quantile (Supplementary Figure 9B) (31).

The FMT intervention displayed different effects on the taxa abundance between the patients (Figure 3E). Patient 1 showed an increase of 13 amplicon sequence variants (ASV) of the genera *Alistipes, Oscillibacter, Coprococcus, Clostridium IV*, and the families Ruminococcaceae and Lachnospiraceae, the order Clostridiales, and the Firmicutes phylum (Figure 3E). Contrary, nine ASV from the genera *Victivallis, Alistipes, Roseburia, Prevotella, Ruminococcus, Blautia*, and *Faecalibacterium* and two ASV of the family Lachnospiraceae decreased after FMT (Figure 3E). Patient 2 showed an increase of ASV of the Ruminococcaceae family, including *Ruminococcus E*, and one ASV of the species *Flavonifractor plautii* (Figure 3E), which has been reported to be increased in MDD-patients (16). Patient 2 showed a decrease of five ASV of the genera *Ruminococcus, Alistipes, Bifidobacterium, Oscillibacter*, and the family Lachnospiraceae (Figure 3E). Further results are documented in the Supplementary Results.

## Discussion

Four weeks after the intake of oral frozen FMT-capsules, depressive symptoms improved in both patients, objectively graded from severe/moderate to mild depression (33); this is in line with previous results implicating that FMT reduced depressive symptoms in patients with irritable bowel syndrome (IBS) 4 weeks after treatment (34). Intriguingly, in the study by Kurokawa et al. (34), the clinical improvement after FMT was accompanied by increased gut bacteria diversity and decreased GI-symptoms. The relationship between IBS and depression seems to be bi-directional (34, 35). Both of our patients were not formally diagnosed with IBS, but experienced GI-symptoms at baseline. While patient 1 was able to defecate regularly even 8 weeks after FMT, patient 2 was initially relieved, but after 4 weeks, again afflicted by constipation.

At baseline, both subjects showed a Ruminoccus enterotype bacterial composition and low fecal moisture, a proxy of slow transit time, and congruent with the prevalence of constipation in mood disorders (36–38). Patient 1 increased in diversity and maintained the Ruminococcus enterotype, indicating a better microbial response to FMT. However, the improvement of constipation in patient 1 is not reflected by the enterotype, since Ruminococcus is associated with slow transit time (36, 37).

Compared to patient 2, patient 1 showed a higher abundance of short-chain fatty acid (SCFA) producers such as *Butyrivibrio* and *Faecalibacterium* that, along with *Dialister*, seem to be depleted in depressed people (16). Additionally, patient 1 showed an increase of species related to other healthy commensal species from the genera *Methanobrevibacter* and *Sporobacter*. Such species are related to low transit time and a healthy microbial establishment indicating the good recovery of the microbial community after oral frozen FMT-capsules. Moreover, patient 1 showed an increase and decrease of different ASV of the *Alistipes* genera after FMT. It is reasonable to assume that different *Alistipes* species may have different roles in host health; it has been reported that the decrease in *Alistipes* exerts an immunoregulatory effect and contributes to the decrease in SCFA which are suggested to have anti-depressant effects (39, 40). Simultaneously, *Alistipes* are increased in depressed subjects (41).

Patient 2 still experienced constipation after the oral frozen FMT-capsules. It has been reported that prolonged constipation leads to a dysbiotic microbial configuration (42, 43); indeed, patient 2 showed increased fecal calprotectin and switched to the Bacteroides-2-enterotype. Although until now this enterotype has mostly been linked to fast transit, constipated Bacteroides-2-individuals do exist (Raes, unpublished results). This patient's bacterial community showed an increase of species of the Flavonifractor genus, which is related to depression (16, 31, 44), and an increase of species of the Streptococcus genus, which is associated with high calprotectin and pro-inflammatory conditions (31). We assume that prolonged constipation in depressed people may have compromised the effectiveness of the oral frozen FMT-capsules by preventing the engraftment of the healthy microbial commensal species. A previous case report of FMT, as mono-treatment for depression and introduced via colonoscopy, reported a MDD-patient who also suffered from constipation (26). Interestingly, 4 days after FMT, the patient's GI- and depressive symptoms improved and persevered until 6 months after FMT (26).

The positive depression outcome did not persevere for both of our patients. Objective rating of the first patient's depressive condition, who improved GI conditions, indicated moderate depression at 8-week follow-up. Contrary, patient 2, who did not improve greatly on GI conditions, remained within the range of mild depression with a tendency to increase depressive symptoms. A study recently reported significant improvement of QoL and fatigue in IBS-patients 3 months after receiving FMT (45), which implicates a long-lasting positive effect of FMT compared to our results. They also found dose-dependent effects and that improvement in QoL and fatigue was not entirely in line with improvement of GI symptoms. FMT results on depressive and GI symptoms are conflicting, and RCTs investigating FMT in depressed patients are lacking. While some studies found improvement in depressive symptoms and QoL after FMT (34, 45), there is also evidence of QoL and depressive symptoms not being affected by FMT (46).

One important factor for such mixed results is the general heterogeneity of illness presentation in MDD and IBS populations. As FMT success might depend on the recipient's microbial composition before FMT or on the microbial resemblance of the donor and the recipient, identifying subgroups of depressed patients might be crucial (47). Another reason could be the differing methodology between studies, such as choosing one (super)donor (45) or several donors (46), the FMT administration (e.g., oral capsule or colonoscopy) (48, 49), or the formulation (e.g., frozen or fresh) (24, 50). Other arising questions regard the optimal dose and durability of FMT (45). Barbara and Ianiro (50) discuss such issues of FMT methodology.

Notably, although the small participant number precludes statistical group comparison, the HAMD-scores of the two FMT and the two placebo-patients present interesting descriptive results. We would expect an improvement in depressive symptoms over time as the patients received TAU. Nonetheless, the two placebo participants presented only a 2-week improvement after placebo-capsule intake, which then relapsed to baseline scores. This might be attributed to the placebo effect, especially as one of the placebo participants thought she had received the active product. The other placebo patient reported increased depressive symptoms, which even restrained her from coming to the post-intervention assessment. Combined with the results from the FMT-patients, these results indicate that frozen oral FMT-capsules as add-on therapy might have the potential to improve depressive symptoms.

Critically, the report of cases suffering from SAEs after FMT raises the importance of extensive donor screening and cautious selection of FMT-patients (50). To overcome these issues, more large-scale controlled clinical studies are needed, investigating gut microbiota modulation in depression, gaining knowledge of its underlying mechanisms, neuroactive potential and beneficial, and harmful microbes and eventually, reconstituting microbes in the laboratory. This would make safety control, retraceability, and substantial FMT production possible (50). The resulting clinical trials could greatly improve our knowledge and eventually lead to the translation of controlled and specific FMT to clinical practice, and finally, improve depressed people's wellbeing.

### Limitations

The current study reports some limitations, starting with the limited sample size. Second, both patients also had comorbidities, such as obesity and constipation, which both might be confounding factors as overweight and constipation seem to be associated with altered gut microbiota composition (51, 52). They received FMT additionally to TAU which makes attribution of effects solely to FMT impossible; the outcome could be influenced by other pathologies or be the result of FMT and antidepressants working in synergy as antidepressants may influence the gut microbiota (53). Additionally, our patients were constipated at baseline, which might be due to medication as some of the medication might modify the transit time (Supplementary Table 4). Third, we did not include any information about the patients' diets. However, diet has been found to be associated with depression (54) and is one of the most significant modulators of gut microbial community (55). A fourth limitation is the comparably low amount of donor stool (8.25 g). As the dose may play a crucial role in the effects, future research should also include dose-finding strategies. Further, although a previous study revealed that oral FMT-capsules are non-inferior to colonoscopy in efficacy in *CDI*-patients (25), the most efficient delivery mode in patients with depression needs to be established.

Lastly, the microbial resemblance of the donor and recipient may play an important role. As we do not have information on the donors' microbial composition, such comparison was impossible in this report. Future studies should include information on the donors' microbiome and compare it to the recipients' microbiome to identify subgroups for better treatment options.

## Patients' Perspectives

Both patients were positive toward the intervention and had a feeling that they had received the active product. They felt better regarding their depressive symptoms, which is visible by the subjective measurement with the BDI-II (Figures 2E,F). In 2021 we contacted the patients via telephone, which was ~2.5 and 1.5 years after the intervention for patient 1 and 2, respectively. At that time, both were going on in their daily lives. One participant emphasized that one of her major treatment milestones was FMT.

## Data Availability Statement

The raw data supporting the conclusions of this article will be made available by the authors, without undue reservation.

## Ethics Statement

The studies involving human participants were reviewed and approved by Ethikkommission Nordwest- und Zentralschweiz. The patients/participants provided their written informed consent to participate in this study. Written informed consent was obtained from the individual(s) for the publication of any potentially identifiable images or data included in this article.

## Author Contributions

AS: had full access to all the data in the study and takes responsibility for the integrity of the data and the accuracy of the data analysis and study supervision. LM, CB, SBo, AS, and UL: study concept and design. JD, JV-C, ACS, NS, CK, ES, GY, JR, and AS: acquisition, analysis, or interpretation of data. JD, JV-C, and AS: drafting the manuscript. All authors: critical revision of the manuscript for important intellectual content. JD and JV-C: statistical analysis. SBo, AS, and UL: obtained funding. LM, SBr, SBo, JR, AS, and UL: administrative, technical, or material support. All authors contributed to the article and approved the submitted version.

## Funding

This work was supported by the Gertrud Thalmann-Fonds (SBo, UL), Seerave Foundation (UL), Kämpf-Bötschi Stiftung (UL), and Research Fund Junior Researchers of the University of Basel (Appln 3MS1041, AS). JV-C was supported by the postdoctoral fellowships fromthe Research Fund–Flanders (FWO 1236321N). The Raes lab was supported by VIB, KU Leuven, and the Rega Foundation.

## Conflict of Interest

The authors declare that the research was conducted in the absence of any commercial or financial relationships that could be construed as a potential conflict of interest.

## Publisher's Note

All claims expressed in this article are solely those of the authors and do not necessarily represent those of their affiliated organizations, or those of the publisher, the editors and the reviewers. Any product that may be evaluated in this article, or claim that may be made by its manufacturer, is not guaranteed or endorsed by the publisher.
