# Supplementary material for: Fecal Microbiota Transplantation (FMT) as an Adjunctive Therapy for Depression—Case Report
Source: Front Psychiatry. 2022 Feb 17;13:815422. doi: 10.3389/fpsyt.2022.815422 (PMC8891755; doi:10.3389/fpsyt.2022.815422)
Supplement: Supplementary file 1 [file Data_Sheet_1.docx]

Supplementary Case Description

Patient 1 was a 53-year-old Caucasian female whose diagnosis comprised MDD and chronic constipation. According to the patient, her first depressive episode started in adolescence, with a suicide attempt later in life. She was first diagnosed with MDD in 2006 and reported no continuous period above two months without depressive symptoms since then. The patient had been hospitalized twice in her life. Depression is common in her intermediate family, with two male second-degree relatives who have suffered from it, one deceased from suicide. At the baseline assessment, the patient had been in stationary therapy for almost three months and treated for depression and constipation (for medical treatment details, see Supplementary Table 1). Nevertheless, depressive symptoms persisted and had increased in their severity. They included "feeling a great inner bleakness", "black thoughts" and "psychological pain".

Also stagnating at a deficient level was the patient's chronic constipation, which had never been alleviated during her life. Despite consulting a dietary coach, using various laxatives, and conducting enemas, defecation was only possible once every two weeks.

Patient 2 was a 58-year-old Caucasian woman with a diagnosis of recurrent MDD. According to the patient, she cannot remember her childhood. As a young woman, she was told that she had been sexually abused as a child. She cannot actively recall being abused; however, her life has been filled with psychological abuse within her family and depression ever since she can remember. She was first diagnosed with depression in 1980 and has been hospitalized twice since then. A family history of depression is not known. After more than two months of in-patient treatment, the patient's symptoms persevered. Her mood was low, and she suffered from dissociation. During that time, whenever any negative emotions appeared, she would dissociate, switch to childlike behavior and sometimes suffer from a panic attack. The patient was medicated with antidepressants and benzodiazepines (for details, see Supplementary Table 2). At that point in time, the patient was suffering from a negative affective state and GI symptoms, such as flatulence and constipation.

**Supplementary Methods**

***Inclusion Criteria:***

1. Age ≥ 18, (2) Body Mass Index (BMI) 18.5 – 40 kg/m^2^
2. Able to provide signed and dated informed consent
3. Hamilton Depression Rating Scale - 17(HAMD - 17) > 17 (1)
4. The patient receives treatment as usual (TAU) for depression
5. Inpatients and outpatients of the UPK

***Exclusion Criteria:***

1. Comorbid psychiatric disturbances such as substance abuse, bipolar disorder, schizophrenia and eating disorders led to the exclusion of participation
2. The same accounted for current medical conditions (i.e. infectious disease), dietary restrictions (also any deviation from UPK standard meals)
3. Recent use of medication aside from standard depression care (i.e. antibiotics)
4. Anticipated use of antibiotics in upcoming four weeks
5. Pregnancy
6. Inability to read and understand the participant's information and informed consent
7. Inability or unwillingness to swallow capsules
8. Active vomiting
9. Intestinal conditions: known or suspected megacolon and/or known small bowel ileus, major gastrointestinal surgery within three months before enrolment (except appendectomy or cholecystectomy), history of total colectomy or bariatric surgery
10. Concurrent intensive chemotherapy, radiation therapy or biological treatment for active malignancy
11. Life expectancy of < 6 months

***Study Design***

The initial study was conducted using a double-blind, placebo-controlled, randomized parallel-group design. The trial was approved by the local ethics committee (Ethikkommission Nordwest- und Zentralschweiz) and was conducted in accordance with the principles of the Declaration of Helsinki and the International Conference on Harmonization Tripartite Guideline on Good Clinical Practice. All patients provided written informed consent. The study was registered at ClinicalTrials.gov prior to study start (NCT03281004). Measurements of depressive and gastrointestinal symptoms took place before the intervention (baseline) as well as four weeks after the intervention (post-intervention). During the four weeks after the intervention weekly assessments took place. A follow-up was conducted after eight weeks. For one of the patients, stool samples were available eight weeks after the intervention. For the other patient, stool samples at follow-up were not available.

While our RCT was running, the Food and Drug Administration (FDA) released a safety alert regarding FMT (https://www.fda.gov/safety/medical-product-safety-information/fecal-microbiota-transplantation-safety-alert-risk-serious-adverse-events-likely-due-transmission). Serious adverse events (SAEs) were observed in six patients with *rCDI*, who received FMT capsules from the same stool bank as we did. Although the participants of our RCT did not report any SAEs, we decided to abort the study for safety reasons. Since the follow-up assessment was around the time that the initial RCT was aborted and the patient has had difficulties with the stool sampling method, we decided not to demand a follow-up sample from this patient. Both patients provided written informed consent

*Randomization and Masking*

Patients were randomly assigned in a 1:1 ratio to receive FMT or placebo capsules. The randomization schedule was produced by an independent researcher, held centrally and not divulged to anyone involved in the trial. A unique treatment number was used to identify each carton of the investigational medicinal product; FMT and placebo cartons were identical in appearance. After randomization, patients were allocated an investigational medicinal product pack in sequential treatment number order. Patients were unaware of whether they received the active product or the placebo and were informed about their allocation after study completion.

*Intervention*

Patients were administered 30 FMT or placebo capsules under the observation of a physician. The choice of 30 capsules was based on prior assessment amongst depressed patients, who expressed a preference for capsules and on existing experience of stool volumes delivered via colonoscopy for *CDI* and some dose-finding research, indicating that low-dose (30 capsules) and high dose (60 capsules) resulted in statistically similar resolution of GI symptoms in *CDI* patients (2). The effect of administering frozen fecal microbiota or FMT capsules appear to be similar to fresh fecal microbiota transplantation in *rCDI* treatment (3, 4). The drug substance contained frozen fecal microbiota, filtered to 330 microns, mixed with glycerol and saline. Each 30-capsule-dose consisted of approximately 8.25g of donor stool. It originated from a single donor within one collection window and was kept frozen either in a -80° Celsius freezer or on dry ice. The stool donor for patient 1 was not the same as for patient 2. The capsules are coated with an internal enteric coating, allowing for stability. Further, they are externally coated in an enteric polymer, allowing for targeted delivery of viable microbial communities to the colon. The placebo capsules were identical in appearance to active capsules but did not contain fecal samples. Placebo capsules contained an autoclaved solution of glycerol and saline, enclosed in an identical gelatin capsule as the active product, including the same enteric polymer coating. Here we report two patients who received the active product.

***Microbiome Analysis***

**DNA extraction and sequencing**. The fecal DNA was extracted following the protocol described in (5). Summarily, DNA was extracted from 150-200mg of the frozen samples using MagAttract PowerMicrobiome DNA/RNA KF kit (QIAGEN) following the manufacturer's instructions. The V4 region of 16S rRNA genes was amplified using the 515F /806R primer pair and purified using the QIAquick PCR Purification Kit. Sequencing was performed using the Illumina MiSeq platform (MiSeq Reagent Kit v2).

**16s rRNA data processing**. Amplicon data from the 16S rRNA gene was analyzed following the DADA2 pipeline specifications (Callahan et al., 2016) ⁠(Briefly, the first 30bp were removed, and the sequence length was set to 130bp and 200bp for the forward and reverse strands, respectively. The sequence error rate, dereplications, the inferred composition of the sample, and the chimaera removal were done using the DADA2 default parameters. The taxonomic assignation was done utilizing the DADA2 RDP implementation (R packages “dada2” function “assignTaxonomy”) using as reference the rdp_train_set_16 (<https://zenodo.org/record/801828#.Xe-PctF7mQc>), similarly the amplicon sequence variant (ASV) annotation was done using the [GTDB_bac120_arc122_ssu_r202_Species](https://zenodo.org/record/4735821/files/GTDB_bac120_arc122_ssu_r202_Species.fa.gz?download=1) trainset (R packages “dada2” function “addSpecies”). The relative abundance was presented at the (ASV) level and summarized to the genus level.

**Microbial load measurement by flow cytometry.** The microbial load of the study cohort was measured as described previously (6). Briefly, 200-250 mg frozen (-80°C) fecal aliquots were diluted in saline solution (0.85% NaCl; VWR International, Germany) and filtered using a sterile syringe filter (pore size of 5 µm; Sartorius Stedim Biotech GmbH, Germany). Next, 1 mL of the microbial cell suspension obtained was stained with 1 µL SYBR Green I (1:100 dilution in DMSO; Thermo Fisher Scientific, Massachusetts, USA) and incubated for 15 min in the dark at 37°C. The flow cytometry analysis was performed using a C6 Accuri low cytometer (BD Biosciences, New Jersey, USA) based on Prest et al. (7). Fluorescence events were monitored using the FL1 533/30 nm and FL3 >670 nm optical detectors. The BD Accuri CFlow software was used to gate and separate the microbial fluorescence events on the FL1/FL3 density plot from the fecal sample background. A threshold value of 2000 was applied on the FL1 channel. Based on the exact weight of the aliquots analyzed, cell counts were converted to microbial loads per gram of fecal material.

**Quantitative microbiome profiling.** The quantitative microbiome profiling (QMP) matrix was built as described by Vandeputte and colleagues (6).

In brief, samples were downsized to even sampling depth, defined as the ratio between sampling size (16S rRNA gene copy number-corrected sequencing depth) and microbial load (the average total cell count per gram of frozen fecal material). 16S rRNA gene copy numbers were retrieved from the rRNA operon copy number database rrnDB33.

**Fecal moisture content.** The fecal moister content was determined as the percentage of mass loss after lyophilization from 0.2 g frozen aliquots of non-homogenized fecal material as previously done in (5).

**Fecal calprotectin measurement.** Fecal calprotectin concentrations were determined using the fCAL ELISA Kit (Bühlmann). The measurements were done on frozen fecal material (-80°C).

**Diversity analysis.** Diversity analysis was performed using the R statistical software (v3.6.3). The beta diversity analysis from the 16S rDNA amplicon sequence variant (ASV) data was estimated by the free statistical package R (v3.6.3). The Bray-Curtis index (library "Vegan", function "vegdist") was used to estimate the dissimilarities between samples in the QMP even sampling depth ASV table. The low frequent ASV (80% of zero data) were removed previous to the dissimilarity estimation. A distance-based redundancy analysis (dbRDA) (library "Vegan" function "capscale") was performed to reduce dimensionality in the taxonomic and functional distance matrix. The Permutational Multivariate Analysis of Variance Using Distance Matrices (ADONIS test) (library "vegan" function "adonis") clinical and metadata variables. The clinical meta-data variables were correlated into the ordination using the function envfit (library "vegan" function "envfit"). Data were hierarchically clustered using the ward.D method (library "stats" function "hclust") and visualized into a heatmap (library "gplots" function "heatmap.2"). The adonis and envfit p-values were adjusted using the Benjamini-Hochberg method (library "stats" function "p.adjust").

The observed richness, the Shannon and the Inverse Simpson index (library "phyloseq" function "estimate_richness") and Pielou's evenness (library "microbiome" function "evenness") were estimated at the genus level for each of the samples of the cohort.

**Enterotyping.** The 16s rRNA bacterial profiles were collapsed at the genus level and integrated along with the FGFP cohort as done in the previous work (5). The Identification of the enterotypes was accomplished with the Dirichlet-multinomial Model (DMM) approach in R (library "DirichletMultinomial" function "dmn").

***Statistical analysis of the microbiome***

The multivariable association between the clinical data and the bacterial taxa were estimated using a negative binomial mixed-effect model (MEM) (library "lme4" function "glmer.nb"), here the dependent variable was set as the abundance of the ASV, the fixed effect was the time variable, and the random effect part of the model was the subject ID. Similarly, the alpha diversity indices, as well as the clinical metadata variables, were model using a mixed-effect model (library "lme4" function "lmer"). The significance of the coefficients was measured by performing the type III ANOVA test over the residual sum of squares (library "car" function "Anova"). The differences between the subjects in the analysis were determined by applying DESeq2 into the ASV even sample depth matrix. The taxonomic summarization at the Phylum level was visualized into a barplot (library "ggplot").

The between-group comparisons of continuous variables were analyzed using Wilcoxon signed rank-sum test (library "stats", function "wilcox.test"). All p-values were adjusted using Benjamini-Hochberg's correction (library "stats", function "p.adjust").

The visualization of the bar plots and principal, boxplots, PcoA were done using the ggplot package.

***Statistical analysis of mapped data***

As the present results come from two cases and do not allow for statistical comparison as in RCTs, we mapped our HAMD change scores together with meta-analysis results from Kirsch and colleagues (2008), which includes 35 trials investigating antidepressant treatment (8), to evaluate our findings in the field of depression treatment (Supplementary Figure 1). We calculated z-scores with RStudio (Version 1.4.1717) and acquired the corresponding p-values to investigate the probability that the change scores of the two cases fit into the (normal)distribution of the patients in the meta-analysis by Kirsch and colleagues (8).

**Supplementary Results**

***Results of our data mapped on data of a meta-analysis***

Contrasting our FMT effects with effect sizes of antidepressant medications (8) revealed that patient 1 had a change score in line with that of antidepressant-medicated patients after four weeks; the change score of 12 appeared to fit into the change score distribution of medicated patients by Kirsch and colleagues (*z* = 0.76, *p* = 0.45). A relapse of symptoms followed (Supplementary Figure 1 and 2) and the eight-week change score of 2 differed from the change score distribution of medicated patients and was significantly lower (*z* = -4.2, *p* < 0.01). Compared to medicated patients in the meta-analysis, patient 2 greatly improved her depressive symptoms after four weeks of the intervention and remained stable until eight weeks follow up (Supplementary Figure 1 and 2). Four-week and eight-week change scores were higher than the change score distribution of the medicated patients in the meta-analysis by Kirsch and colleagues (four-week change score: *z* = 5.23, *p* < 0.01; eight-week change score: *z* = 4.24, *p* < 0.01), indicating greater improvement in our patient 2 compared to the medicated patients of the meta-analysis.


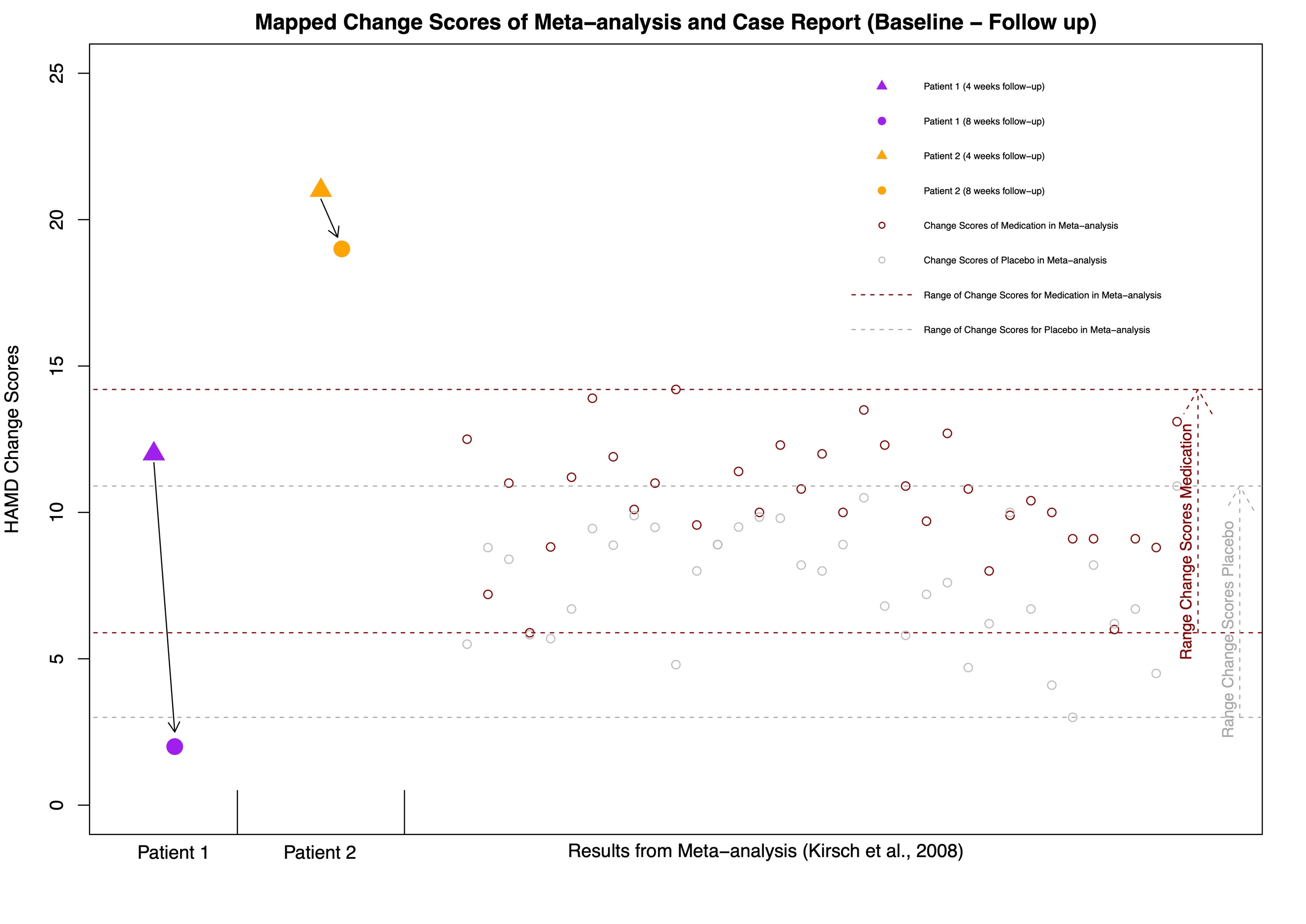


Supplementary Figure 1. Mapped change scores of patients 1 and 2 as well as change scores from Kirsch et al. (2008). Kirsch and colleagues’ meta-analysis included 35 trials, investigating one of four antidepressants: Fluoxetine, Venlafaxine, Paroxetine and Nefazodone. As far as understandable from reported data, trials were of four, five, six- or eight-weeks duration (8). The range of change scores over all four medication treatments is indicted by the red dotted arrow, the range of overall placebo change scores with a grey dotted arrow. Purple symbols indicate patient 1, orange symbols patient 2. Triangle indicates the patients’ change score four weeks post-FMT compared to baseline and the dots mark the change score eight weeks post-FMT compared to baseline. Black arrows illustrate the decrease of change score, which implies an aggravation of depressive symptoms.


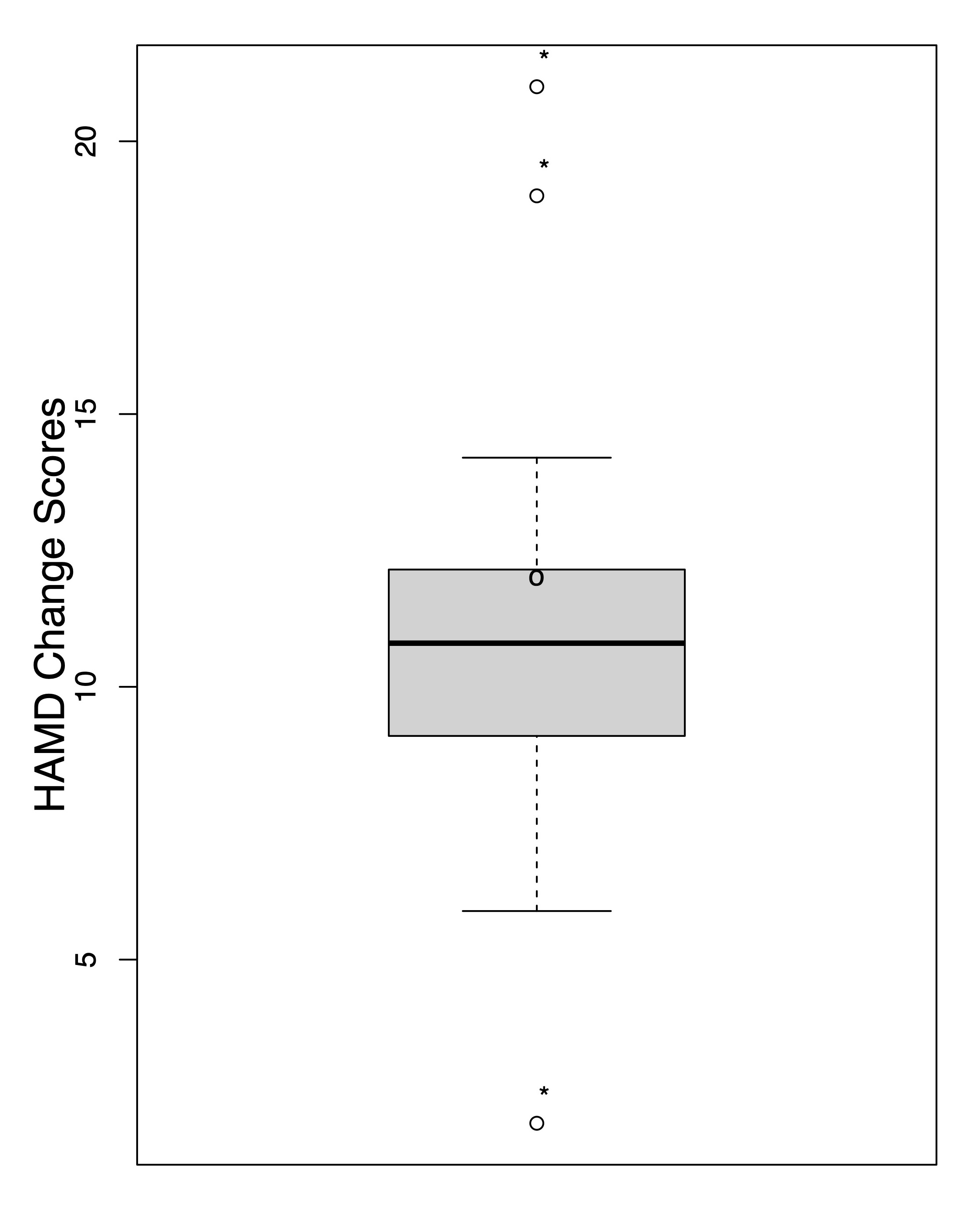


Supplementary Figure 2. Boxplot of HAMD change scores including medicated patients from meta-analysis by Kirsch et al. (2008) as well as the change scores of the two FMT cases (one point four weeks after FMT and one point eight weeks after FMT for each case). Three of the four change scores were outside of the distribution of medicated patients (p < 0.01).

***Results of placebo participants***

HAMD scores for both placebo participants improved in the two weeks after placebo-capsule intake. However, after the second week, symptom scores raised back up to the initial scores at baseline (placebo participant 1 from 16 at baseline back to 16 four weeks later at post-intervention and placebo participant 2 from 22 at baseline to 11 four weeks later at post-intervention). Eight weeks after the intervention, HAMD score for placebo participant 1 decreased to 9 points. HAMD score at follow up for placebo participant 2 is not available for two reasons: the depressive symptoms of this participant increased to a level as that she did not come back for post-intervention assessment. We then measured the HAMD score only by telephone. Additionally, at the timepoint of follow up measurement for placebo participant 2, the study had already been terminated and as we then learned that she had received the placebo, we relinquished to do the follow-up assessment with her.


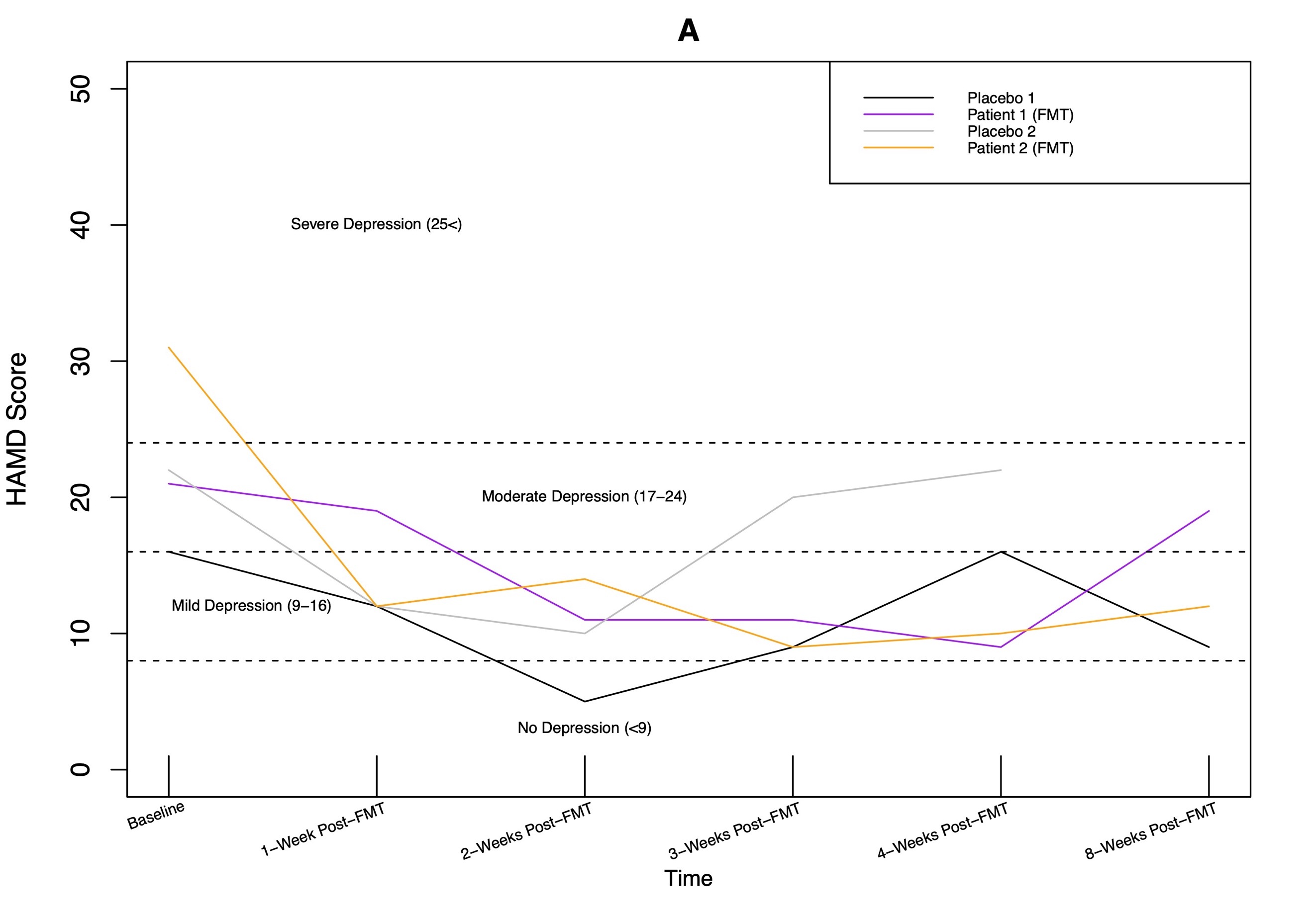


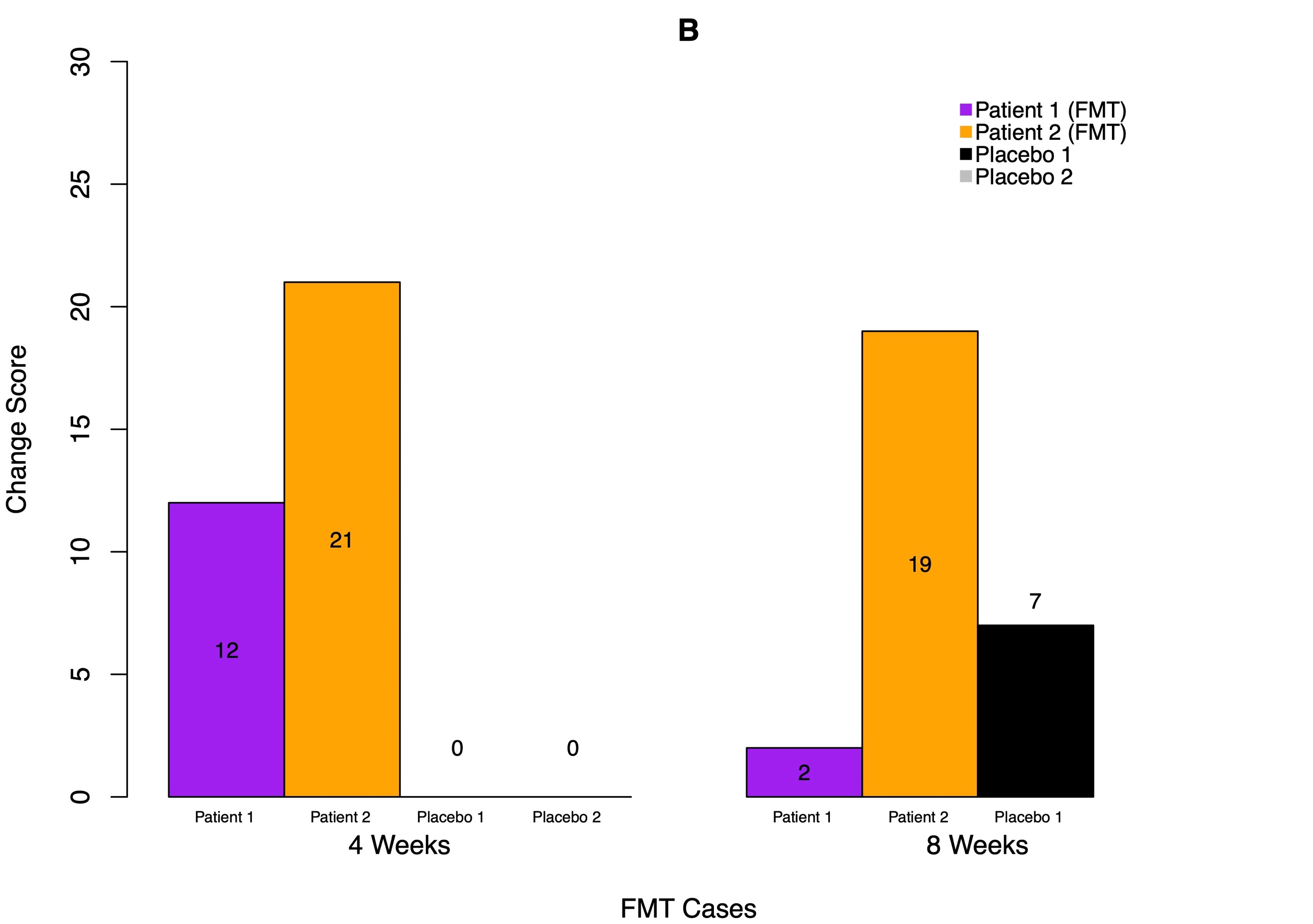


Supplementary Figure 3. A) HAMD scores for all 4 patients that had been included in the study at the time of premature termination of the study. Placebo patients are shown in black and grey. HAMD score at follow-up for placebo participant 2 is not available for two reasons: the depressive symptoms of this participant increased to a level as that she did not come back for post-intervention assessment. We then measured the HAMD score only by telephone. Additionally, at the timepoint of follow-up measurement for placebo participant 2, the study had already been terminated and as we then learned that she had received the placebo, we relinquished to do the follow-up assessment with her. B) Change scores for both FMT patients (patient 1 and patient 2) and for both placebo participants (placebo 1 and placebo 2). As change scores were calculated by subtracting the score at post-intervention from the score at baseline, a higher change score indicates lower depressive symptoms score. Again, the follow-up score for placebo participant is not available.

***Results of the bacterial taxa responding to FMT***

Overall, microbiota composition in these two patients resembles that of the western population; *Feacalibacterium*, *Blautia*, and *Anaerostipes* were among the most abundant bacteria (Supplementary Figure 4 & B).

Over the whole dataset, microbial communities tended to cluster more according to participant identification number rather than time point, yet no significant effect was found for either variable (Supplementary Figure 5 B), Supplementary Table 3; Adonis adjusted *p* = 0.226).

The Deseq2 differential abundance analysis identified 46 different amplicon sequence variants (ASV) that were differentially enriched between the participants (Supplementary Figure 5 A) (DESeq2 adjusted *p* < 0.05). Patient 1 showed an increase of archaea and bacteria species abundant in low-transit-time bacterial communities, such as the phylum Euryarchaeota and the genus Methanobrevibacter (5) (Supplementary Figure 4 B and Supplementary Figure 5 A); this is congruent with the observed moisture level of the Ruminocuccus enterotype (9). Similarly, the patient showed an increase of ASV of the genera *Methanobrevibacter*, *Butyrivibrio*, *Sporobacter*, *Olsenella*, *Slackia, Faecalibacterium*, and *Dialister* (Supplementary Figure 5 A) (DESeq2 adjusted *p* < 0.05).

Patient 2 showed an increase of ASV of the species *Bifidobacterium kashiwanohense*, and of the genera *Ruminococcus* and *Roseburia*, *Eubacterium*, *Subdoligranulum*, *Flavonifractor*, *Bilophila* and, *Streptococcus* (Supplementary Figure 5 A).

*
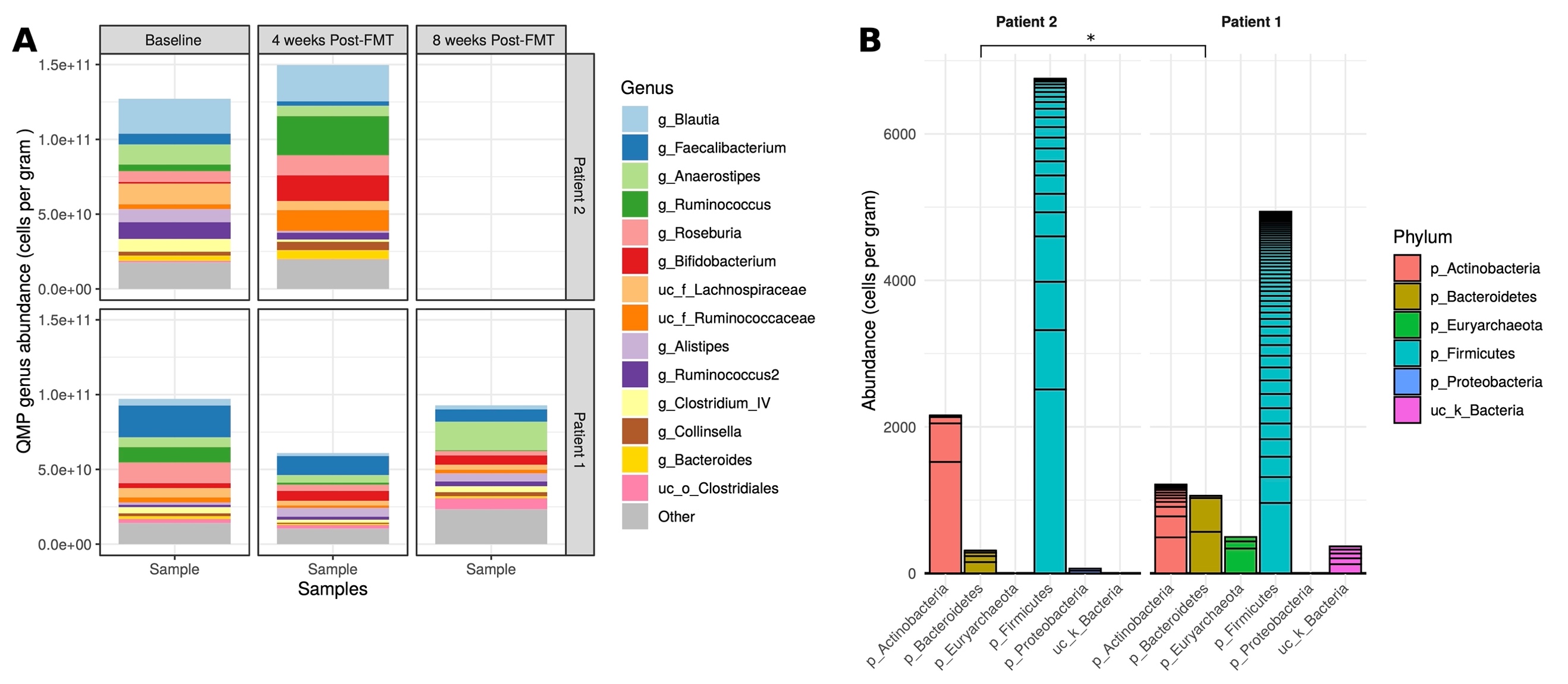
*Besides differences, there were also similarities of FMT effects in these two patients. Common ASV changes were defined as such when both the continuous and discrete mixed-effect-model time coefficients were significant (adjusted *p* < 0.1) (Figure 3 E) and showed the same trend for both trial subjects. We found that the FMT increased the abundance of ASVs of the genera *Bifidobacterium*, *Blautia*, and the family Lachnospiraceae in both patients (Figure 3 E). Contrary, nine ASV of the genera *Eubacterium E, Coprococcus, Faecalibacterium, Butyricicoccus, Ruminococcus,* and *Bacteroides* and species from the Ruminococcaceae and *Lachnospiraceae* families such as *Lachnospira eligens*, were reduced (Figure 3 E).

Supplementary Figure 4. Microbiota communities of the 2 patients on A) genus level and B) phylum level.


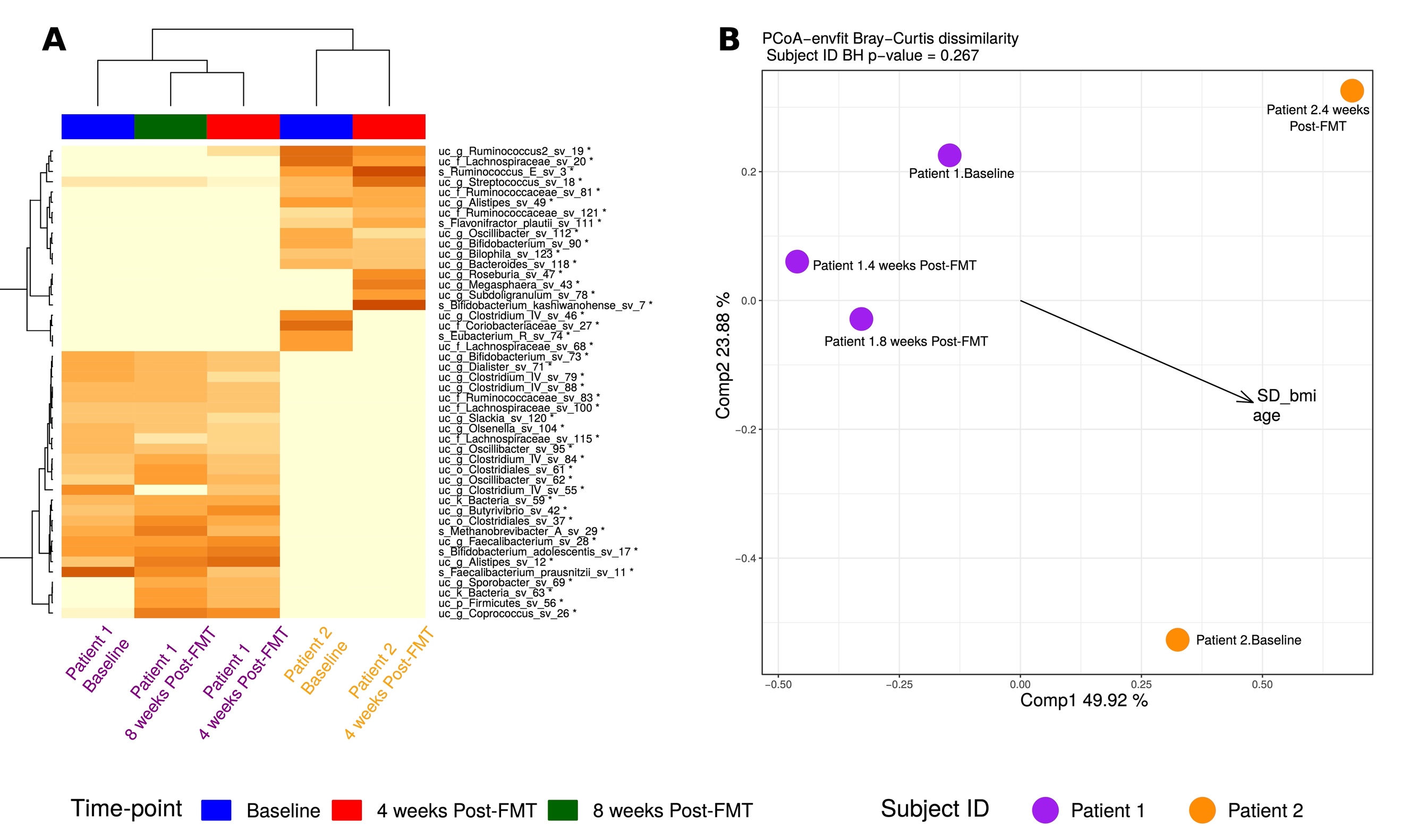


Supplementary Figure 5. A) Hierarchical clustering patients and time points using the ASV-QMP matrix based on Bray-Curtis dissimilarity and the ward.D clustering criteria. The clustering considered all the ASV, but only those significant according to the DESeq2 method (adjusted p-value < 0.05 and |effect size| > 2) were represented in the heatmap and marked with an asterisk. B) Bray-Curtis principal coordinates analysis. The arrows represent the envfit metadata covariation with the two principal components.

Supplementary Figure 6. Effect of the FMT over the transit time and local inflammation. Mixed-effects models (MEM) of the (A) moisture, (B) calprotectin and C) the bacterial load for both patients. Both measurements were modelled into a discrete manner and represented the results into the boxplot figures and in a continuous way, representing the MEM slope into the line plots. The grey area into the continuous MEM represents the 95% confidence level. The red, blue, and dark green colors represent the different time points. Patients are represented in orange and purple.


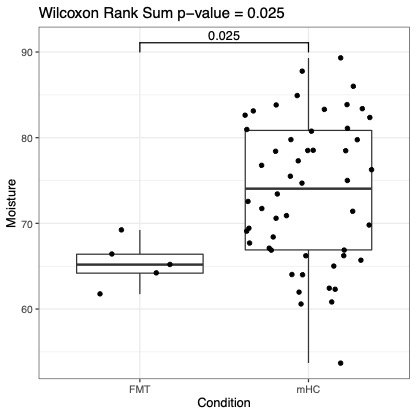


Supplementary Figure 7. Moisture levels of the 2 patients compared to a healthy population.


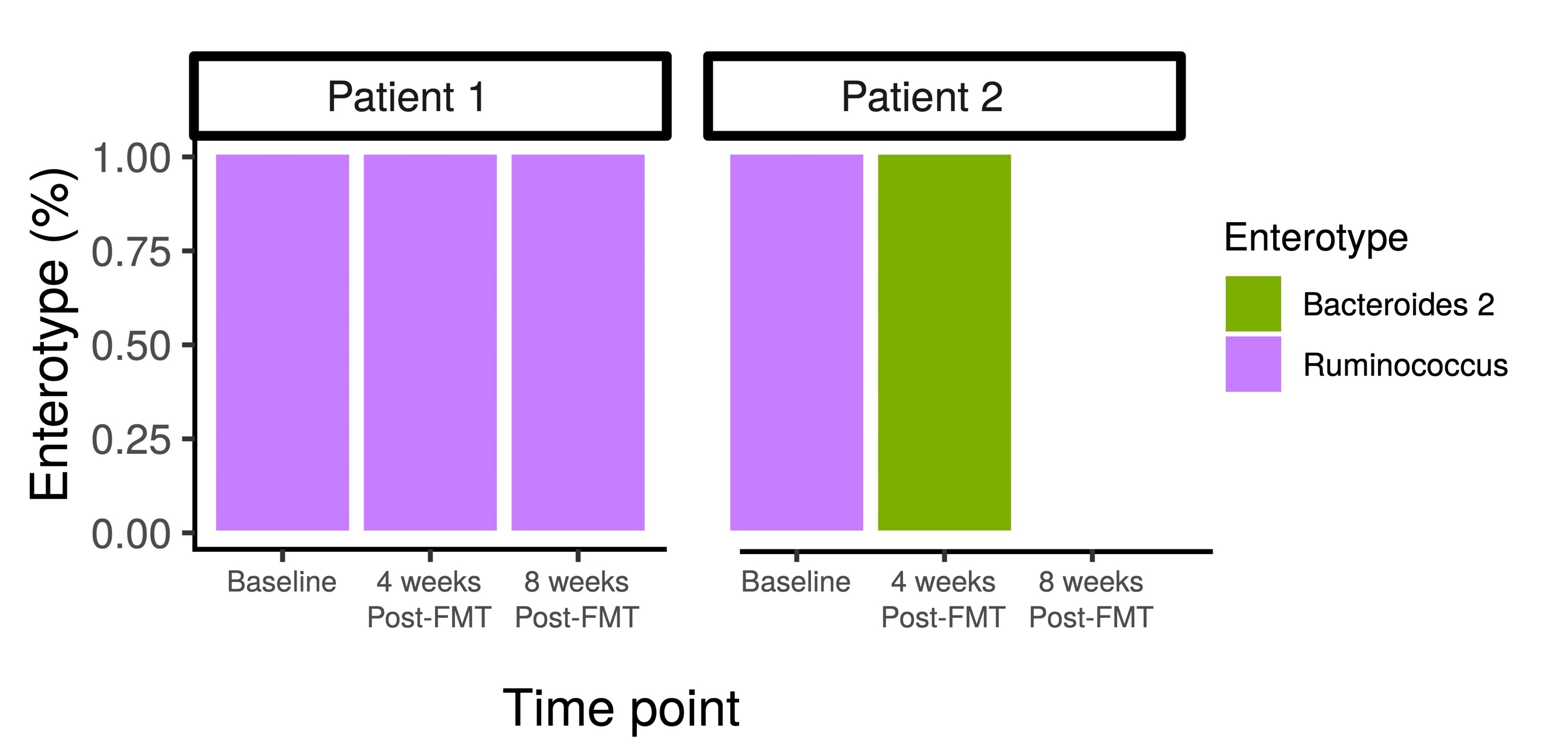


Supplementary Figure 8. Enterotypes of the 2 patients over the available time points.


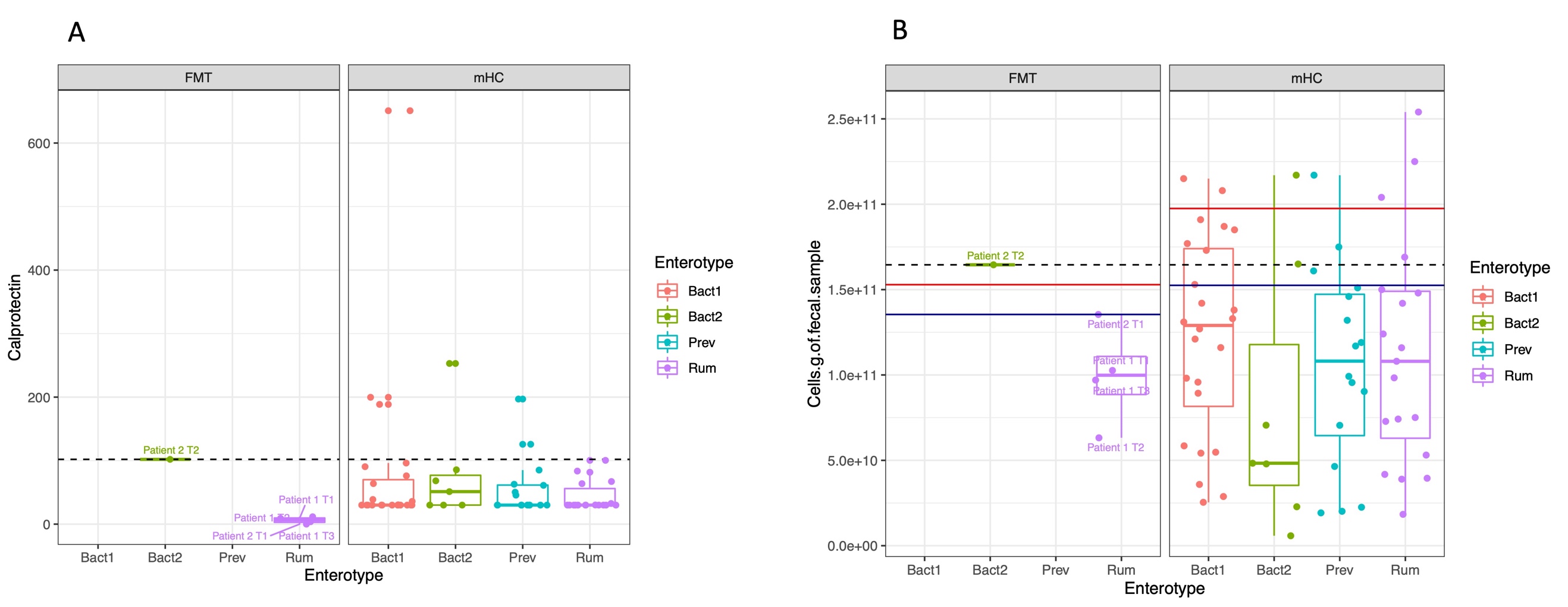
Supplementary Figure 9. A) Fecal calprotectin levels in our FMT cases compared healthy subjects. B) Cell counts in our FMT cases versus healthy subjects.

| Supplementary Table 1. Medication of patient 1 | | | | | | | | | |
| --- | --- | --- | --- | --- | --- | --- | --- | --- | --- |
| Active Ingredient | Brand Name | Type | Time Period | | | | | | |
|  |  |  | 28.11.18- 12.12.18 | 13.12.18- 18.12.18 | 19.12.18- 20.12.18 | 21.12.18- 26.12.18 | 27.12.18- 08.01.19 | 09.01.19- 11.01.19 | 12.01.19- 31.01.19 |
| Lamotrigin 250mg | Lamictal | Anticonvulsants | 1 | 1 | 1 | 1 | 1 | 1 | Discharged |
| Trazodon 100mg | Trittico | Antidepressant (SSRI) | 2.5 | 2.5 | 2.5 | 2.5 | 2.5 | 2.5 | Discharged |
| Macrogol 13.125g, Natrium chloride 350.7mg, Natriumhydrogencarbonate 178.5mg, Potassium chloride 46.6mg | Movicol | Laxative | 3 | 0 | 0 | 0 | 0 | 0 | Discharged |
| Psyllium 491.5mg | Metamucil | Laxative | 3 | 0 | 0 | 0 | 0 | 0 | Discharged |
| Bupropion 150mg | Wellbutrin | Antidepressant (NDRI) | 0 | 1 | 0 | 0 | 0 | 0 | Discharged |
| Vortioxetin 5mg | Brintellix | Antidepressant (serotonin modulator and stimulator) | 0 | 0 | 0 | 1 | 2 | 3 | Discharged |

*Note.* Medication for TAU for depression and constipation for patient 1 during eight-weeks post-FMT. The time period is based on changes in medication prescription. The patient was discharged mid-January and medication intake was therefore not traceable. Provisional medication was available, yet not taken during the eight weeks.

| Supplementary Table 2. Medication of patient 2 | | | | | | | | | | |
| --- | --- | --- | --- | --- | --- | --- | --- | --- | --- | --- |
| Active Ingredient | Brand Name | Type | Time Period | | | | | | | |
|  |  |  | 17.02.20- 20.02.20 | 21.02.20- 26.02.20 | 27.02.20- 04.03.20 | 05.03.20- 10.03.20 | 11.03.20- 12.02.20 | 13.03.20- 19.03.20 | 20.03.20- 23.03.20 | 24.03.20-14.04.20 |
| Lamotrigin 100mg | Lamictal | Anticonvulsants | 6 | 6 | 6 | 6 | 6 | 6 | 6 | 6 |
| Trazodone hydrochloride 100 mg | Trittico | Antidepressant | 1 | 1 | 1 | 1 | 1 | 1 | 1 | 0 |
| Escitalopram 10mg | Escitalopram | Antidepressant | 2 | 2 | 2 | 2 | 2 | 2 | 2 | 2 |
| Lorazepam 1mg | Temesta | Benzodiazepine | 2 | 1.5 | 1.5 | 1.5 | 1.5 | 1.5 | 1 | 1 |
| Pregabalin 150mg | Pregabalin | Anticonvulsants | 1 | 1 | 1 | 1 | 1 | 1.5 | 2 | 2 |
| Chlorprothixen hydrochloride 50mg | Truxal | Antipsychotic | 5 | 5 | 5 | 5 | 5 | 5 | 5 | 5 |
| Clotiapin 40 mg | Entumin | Antipsychotic | 0 | 0 | 0 | 1 | 1.5 | 0 | 0 | 0 |

*Note.* Medication for TAU for depression for patient 2 during eight-weeks post-FMT. The time period is based on changes in medication prescription. The provisional medication was available and taken as follows: 30 times Chlorprothixen hydrochloride 50mg, 4 times Chlorprothixen hydrochloride 15mg, 4 times Lorazepam 1mg (Temesta Expidet), 3 times Lorazepam 1mg (Temesta), 1 time Paraffin 1.9g (Paragol; laxative) and 2 times Natrium picosulfat-1-water 7.5mg (Laxoberon; laxative). Additionally, she has received intravenous Ketamine therapy 1/month as pain treatment since 2016.

| Supplementary Table 3. Multivariate analysis of variance results table | | | | |
| --- | --- | --- | --- | --- |
| Variable | Fmodel | R2 | p-value | BH adj p-value |
| Subject ID | 2.577 | 0.462 | 0.100 | 0.267 |
| BMI | 2.577 | 0.462 | 0.100 | 0.267 |
| Age | 2.577 | 0.462 | 0.100 | 0.267 |
| Calprotectin corrected | 1.738 | 0.367 | 0.175 | 0.350 |
| Time point | 0.553 | 0.356 | 0.933 | 0.975 |
| Moisture | 0.776 | 0.205 | 0.733 | 0.975 |
| HAMD | 0.735 | 0.197 | 0.742 | 0.975 |
| GSRS | 0.619 | 0.171 | 0.975 | 0.975 |

*Note.* Statistics of the patient metadata according to adonis multivariate analysis of variance.

| Supplementary Table 4. Medication and side effects on transit time and calprotectin | | | | | |
| --- | --- | --- | --- | --- | --- |
| Active Ingredient | Brand Name | Type | Effect on  transit time | Effect on  Calprotectin | References |
| Lamotrigin 100mg | Lamictal | Anticonvulsants | Diarrhea (Rare) | None | [https://www.drugs.com/lamictal.html; https://www.fda.gov/media/79324/download](https://www.drugs.com/lamictal.html) |
| Trazodone hydrochloride 100 mg | Trittico | Antidepressant | diarrhea, constipation (Less common) | None | [https://www.drugs.com/trazodone.html; https://www.mayoclinic.org/drugs-supplements/trazodone-oral-route/side-effects/drg-20061280](https://www.drugs.com/trazodone.html) |
| Escitalopram 10mg | Escitalopram | Antidepressant | Diarrhea (common*) |  | <https://www.mayoclinic.org/drugs-supplements/escitalopram-oral-route/side-effects/drg-20063707> |
| Lorazepam 1mg | Temesta | Benzodiazepine | constipation (incidence  not known*) | None | <https://www.mayoclinic.org/drugs-supplements/lorazepam-oral-route/side-effects/drg-20072296> |
|  | | | | | |
|  | | | | | |
| Continued from Supplementary Table 4. Medication and side effects on transit time and calprotectin | | | | | |
| Pregabalin 150mg | Pregabalin | Anticonvulsants | diarrhea (rare); severe constipation (Incidence not known) | None | <https://www.mayoclinic.org/drugs-supplements/pregabalin-oral-route/side-effects/drg-20067411> |
| Chlorprothixen hydrochloride 50mg | Truxal | Antipsychotic | None | None | <https://www.wikidoc.org/index.php/Chlorprothixene> |
| Clotiapin 40 mg | Entumin | Antipsychotic | constipation (Incidence not known) | None | <https://www.tabletwise.net/medicine/clotiapine> |
| Bupropion 150mg | Wellbutrin | Antidepressant (NDRI) | Constipation | NA | https://compendium.ch/product/1075670-wellbutrin-xr-ret-tabl-150-mg |
| Vortioxetin 5mg | Brintellix | Antidepressant (serotonin modulator and stimulator) | Constipation, diarrhea | NA | https://compendium.ch/product/1323039-brintellix-filmtabl-5-mg |
|  | | | | | |
|  | | | | | |
| Continued from Supplementary Table 4. Medication and side effects on transit time and calprotectin | | | | | |
| Macrogol 13.125g, Natrium chloride 350.7mg, Natriumhydrogencarbonate 178.5mg, Potassium chloride 46.6mg | Movicol | Laxative | Increase of transit time | NA | https://compendium.ch/product/1080123-movicol-aromafrei-plv/mpro |
| Psyllium 491.5mg | Metamucil | Laxative | Increase of transit time | NA | https://compendium.ch/product/1098398-metamucil-n-mite-plv-5-8-g-orange |

*Note.* *These side effects may go away during treatment as the subject gets adapted to the medicine. NA: not available, to the best of our knowledge, we could not find information on the effect on calprotectin.

References

1. Zimmerman M, Martinez JH, Young D, Chelminski I, Dalrymple K. Severity classification on the Hamilton Depression Rating Scale. J Affect Disord. 2013;150(2):384-8.

2. Fischer M, Allegretti J, Smith M, Klank M, Mendiola G, Vo E. A multi-center, cluster randomized dose-finding study of fecal microbiota transplantation capsules for recurrent Clostridium difficile infection. United Eur Gastroenterol J. 2015;3:561-71.

3. Lee CH, Steiner T, Petrof EO, Smieja M, Roscoe D, Nematallah A, et al. Frozen vs Fresh Fecal Microbiota Transplantation and Clinical Resolution of Diarrhea in Patients With Recurrent Clostridium difficile Infection: A Randomized Clinical Trial. JAMA. 2016;315(2):142-9.

4. Kao D, Roach B, Silva M, Beck P, Rioux K, Kaplan GG, et al. Effect of Oral Capsule- vs Colonoscopy-Delivered Fecal Microbiota Transplantation on Recurrent Clostridium difficile Infection: A Randomized Clinical Trial. JAMA. 2017;318(20):1985-93.

5. Falony G, Joossens M, Vieira-Silva S, Wang J, Darzi Y, Faust K, et al. Population-level analysis of gut microbiome variation. Science. 2016;352(6285):560-4.

6. Vandeputte D, Kathagen G, D'hoe K, Vieira-Silva S, Valles-Colomer M, Sabino J, et al. Quantitative microbiome profiling links gut community variation to microbial load. Nature. 2017;551(7681):507-11.

7. Prest EI, Hammes F, Kötzsch S, van Loosdrecht MC, Vrouwenvelder JS. Monitoring microbiological changes in drinking water systems using a fast and reproducible flow cytometric method. Water Res. 2013;47(19):7131-42.

8. Kirsch I, Deacon BJ, Huedo-Medina TB, Scoboria A, Moore TJ, Johnson BT. Initial severity and antidepressant benefits: a meta-analysis of data submitted to the Food and Drug Administration. PLoS Med. 2008;5(2):e45.

9. Vieira-Silva S, Sabino J, Valles-Colomer M, Falony G, Kathagen G, Caenepeel C, et al. Quantitative microbiome profiling disentangles inflammation- and bile duct obstruction-associated microbiota alterations across PSC/IBD diagnoses. Nat Microbiol. 2019;4(11):1826-31.
